# Energy scavenging from the diurnal cycle with a temperature-doubler circuit and a self-adaptive photonic design

**DOI:** 10.1515/nanoph-2023-0695

**Published:** 2024-01-15

**Authors:** Zheng Zhang, Xiaodong Zhao, Zhen Chen

**Affiliations:** Jiangsu Key Laboratory for Design & Manufacture or Micro/Nano Biomedical Instruments, School of Mechanical Engineering, Southeast University, Nanjing 210096, China

**Keywords:** temperature-doubler, self-adaptive photonic design, fluctuating thermodynamic resources, coupled photonic and thermal optimization

## Abstract

A temperature-doubler circuit is the functional equivalent of a voltage-doubler in the thermal domain. Effective temperature-doubler circuits could benefit energy scavenging from fluctuating thermal resources, e.g. the diurnal cycle. However, the current paradigm relies on static photonic designs of the selective solar absorber or blackbody emitter, which aims at maximizing energy harvesting from either the sun or outer space, but not from both. Furthermore, photonic and thermal optimizations have not yet been coupled to maximize the power output. Here we develop a general framework to optimize the energy acquisition and conversion simultaneously to maximize a temperature-doubler’s power output under a realistic solar-thermal boundary condition. With an ideal self-adaptive absorber/emitter to fully exploit the thermodynamic potential of both the sun and outer space, the theoretical limit of the temperature-doubler circuit’s average output power in a diurnal cycle is found to be 168 W m^−2^, a 12-fold enhancement as compared to the blackbody emitter. We provide a numerical design of such a self-adaptive absorber/emitter, which, combined with a thermoelectric generator, generate 2.3 times more power than the blackbody emitter in a synthetic “experiment”. The model further reveals that, as compared to traditional thermal circuits, the key merit of the temperature-doubler is not to enhance the total power generation, but to convert the fluctuating thermodynamic input to a continuous and stable power output in a 24 h day-night cycle.

## 1 Introduction

Periodic thermodynamic resources, most notably from the diurnal cycle, are widely available but significantly underutilized because of the conflict between the time-variable energy supply of these resources and the steady power requirements of many end-user applications. Previous studies have explored the conversion of these fluctuating resources into fluctuating work using either thermal storage elements to buffer and time shift the fluctuations [1]–[6] or thermal diodes and switches to bias the heat flow [4], [7]–[13], but not both. Inspired by the voltage doubler circuit that converts an AC voltage input into a DC output with magnitude twice as large as that of the AC one, we previously proposed a temperature-doubler circuit to reconcile this conflict [14]. This temperature-doubler circuit employs thermal diodes to extract and hold, and thermal masses to buffer and smooth, both extremes of the fluctuating temperature of the plate, resulting from harvesting the hotness of the sun and the coldness of outer space in a day-night cycle.

Yet this precedent work idealizes the plate as a periodic temperature resource with zero output thermal impedance, which implies unrealistic capability of sourcing and sinking arbitrarily large heat flow. Although a more practical multi-mode solar-thermal boundary condition was briefly considered, it optimized the spectrum of the plate without considering the thermal impedance matching with the temperature-doubler circuit, leading to an output power that is much lower than the theoretical limit. A coupled photonic and thermal design that simultaneously optimizes both the energy acquisition and the energy conversion processes is still lacking.

Another limitation of the precedent work is that the static photonic design of the plate cannot exploit the full thermodynamic potential of the hot sun and the cold outer space. On one hand, a blackbody absorber suffers from heat losses through infrared radiation at daytime [15], [16]; on the other hand, a selective solar absorber with a step-function spectrum cuts off the IR loss at daytime, which, however, hinders heat dissipation through radiative cooling at nighttime [17]–[21]. A self-adaptive photonic design, whose spectrum dynamically switches from a selective solar absorber at daytime to a blackbody emitter at nighttime, could potentially overcome this limitation. Yet none of the previous studies [22]–[26] exploring this idea was optimized to maximize the power output of the temperature-doubler circuit.

To address these shortcomings, we extend our previous framework [14] to optimize the photonic and thermal designs simultaneously. This coupled optimization leads to an optimal thermal resistance of a heat engine that is matched with the resistance of the plate with an optimal spectrum. These two optimum values together determine the upper bound of the power output of the temperature-doubler circuit under the realistic multi-mode solar-thermal boundary condition, which are 168, 49.4, and 14.2 W m^−2^ for the self-adaptive absorber/emitter, static selective solar absorber, and the blackbody emitter, respectively. These analyses further highlight that, as compared to the traditional thermal circuits that do not exploit thermal diodes and masses, the temperature-doubler circuit does not always generate more power output. Instead, its key merit is to drive a heat engine continuously and smoothly over a 24-h day-night cycle. Finally, a vanadium dioxide (VO_2_) based multilayer stack is designed, which automatically switches between a selective solar absorber at daytime and a blackbody emitter at nighttime. With synthetic “experiments”, we demonstrate that this self-adaptive photonic design leads to a 2.3-fold enhancement in power output as compared to the blackbody emitter.

## 2 Results

### 2.1 Concept and theoretical limits

**Figure 1:**
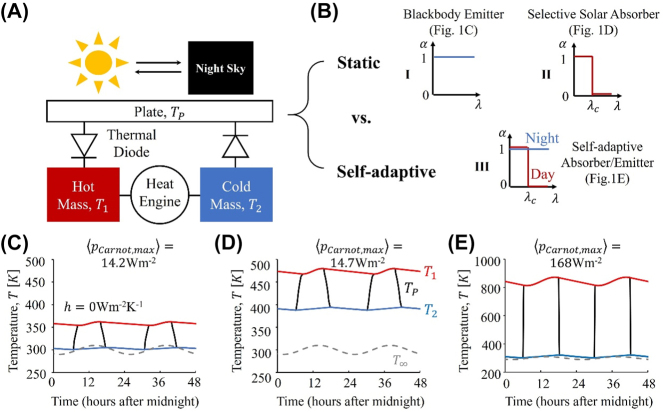
Concept and theoretical limits. (A) Schematic of the temperature-doubler circuit, which uses thermal diodes to extract, and thermal masses to buffer and smooth, both extremes of the periodic temperature of the plate, resulting from the diurnal cycle. (B) Idealized static versus self-adaptive spectra of the plate: blackbody emitter (I), selective solar absorber (II), and self-adaptive absorber/emitter (III). (C–E) Corresponding temperature profiles of the plate and thermal masses, assuming a Carnot heat engine and perfect thermal insulation of the temperature-doubler circuit from the environment (*h* = 0 W m^−2^ K^−1^). Note here temperature profiles result from coupled photonic and thermal designs as discussed in detail in next section and Figure 2. These comparisons highlight that the self-adaptive absorber/emitter combines the merits of the two static spectra, and leads to the maximum output power (normalized by the surface area of the plate) of the temperature-doubler circuit averaged in a diurnal cycle, $\left\langle p_{\mathrm{Carnot,max}}\right\rangle =168$ W m^−2^.

Figure 1A shows the concept of a temperature-doubler circuit [14], consisting of a plate, two thermal masses, two thermal diodes, and a heat engine. From a fluctuating thermal environment, e.g. the alternating of day and night on the earth, this temperature-doubler circuit can generate a near-constant Δ*T*_*Engine*_ = *T*_1_ − *T*_2_, where *T*_1_ and *T*_2_ are the temperatures of the two thermal masses (red and blue lines in Figure 1C–E), to drive a heat engine continuously and smoothly over a 24-h day-night cycle. The essence of this circuit is to use the diodes to extract, and the thermal masses to buffer and hold both extremes of the plate temperature, *T*_*p*_ (black lines in Figure 1C–E), so that now *T*_1_ remains close to the maximum of *T*_*p*_ and *T*_2_ close to the minimum of *T*_*p*_ throughout the cycle.

With three simplified spectra (I, II, and III in Figure 1B), we illustrate limitations of the static photonic designs of the plate and highlight the merit of the self-adaptive design in simultaneously exploiting the full thermodynamic potential of the sun and outer space, thus achieving theoretical limits of the temperature-doubler circuit in a diurnal cycle.

A blackbody emitter (Figure 1B-I) takes full advantage of outer space and dissipates as much heat as possible to minimize the temperature of the plate during the cooling half cycle at night (Appendix C). However, this merit turns into a demerit during the day: the broadband infrared emission significantly reduces the temperature of the plate during the heating half cycle, thus wasting the heating potential of the sun. In contrast, a selective solar absorber (Figure 1B-II) trims the infrared emission after a characteristic wavelength, *λ*_*c*_, and thus takes full advantage of the sun during the heating half cycle during the day. However, this advantage again turns into a disadvantage at night: the lack of infrared emission significantly limits the cooling of the plate at night, thus wasting the cooling potential of outer space.

Is it possible to take advantage of the merits of both spectra above while avoiding their demerits? We notice that the coldness of the nighttime sky occurs many hours out-of-phase from the peak solar heating. To leverage on this fact, an ideal absorber/emitter that drives a temperature-doubler circuit to take full advantage of the two thermodynamic resources, the hot sun and cold outer space, should be self-adaptive: it turns from a selective solar absorber at daytime to a blackbody emitter at nighttime, and vice versa (Figure 1B-III). We note here a blackbody emitter, instead of the “ideal” selective emitter for radiative cooling purpose which has unity emissivity between 8 and 13 μm and zero emissivity elsewhere, is employed at night, because the former always outperforms the latter in this temperature-doubler circuit (Appendix C).

To validate the intuition above, we extend our previous framework to simultaneously analyze the energy acquisition by the plate and the energy conversion by the temperature-doubler circuit through a coupled photonic and thermal optimization. This coupled analysis is necessary because the optimization of the spectrum of the plate alerts the external thermal resistance of the temperature-doubler circuit, which, from the point of view of impedance matching, inevitably requires re-optimization of the internal thermal resistance.

With assumptions and input parameters explained in next section, Figure 1C–E confirm our intuition above: the blackbody emitter lowers the temperature of the cold thermal mass, *T*_2_ (blue in Figure 1C), to approach the ambient temperature, *T*_∞_ (gray), but suffers with a low hot mass temperature, *T*_1_ (red), because of the infrared heat loss during the day. Likewise, the selective solar absorber significantly improves *T*_1_ (red in Figure 1D), but bears a high *T*_2_ (blue) due to the lack of heat dissipation mechanism. As a result, neither of these static spectra harnesses the thermodynamic potential of the sun and outer space simultaneously. In contrast, the self-adaptive absorber/emitter inherits the high *T*_1_ of the selective solar absorber and the low *T*_2_ of the blackbody emitter, thus providing a maximum temperature difference Δ*T* = *T*_1_ − *T*_2_, which, assuming a Carnot engine, generates a maximum output power of 168 W m^−2^, which is normalized by the surface area of the plate and averaged over a 24 h day-night cycle. Although it is not a fair comparison, here, assuming a typical rooftop setup (with parasitic heat transfer coefficient, *h* = 10 W m^−2^ K^−1^), we find the Shockley–Queisser limit, 90 W m^−2^, averaged in a diurnal cycle with the same assumptions and input parameters [27].

### 2.2 Coupled photonic and thermal optimization

We now explain details of the optimization to arrive at the theoretical limits in Figure 1C–E. The optimization involves two key physical processes, energy acquisition from the sun/outer space and energy conversion using the temperature-doubler circuit. While the former relies on photonic design of the plate, the latter requires thermal design of the temperature-doubler circuit. As will be clear from the following analysis, these two optimization processes are correlated and cannot be considered independently.

**Figure 2:**
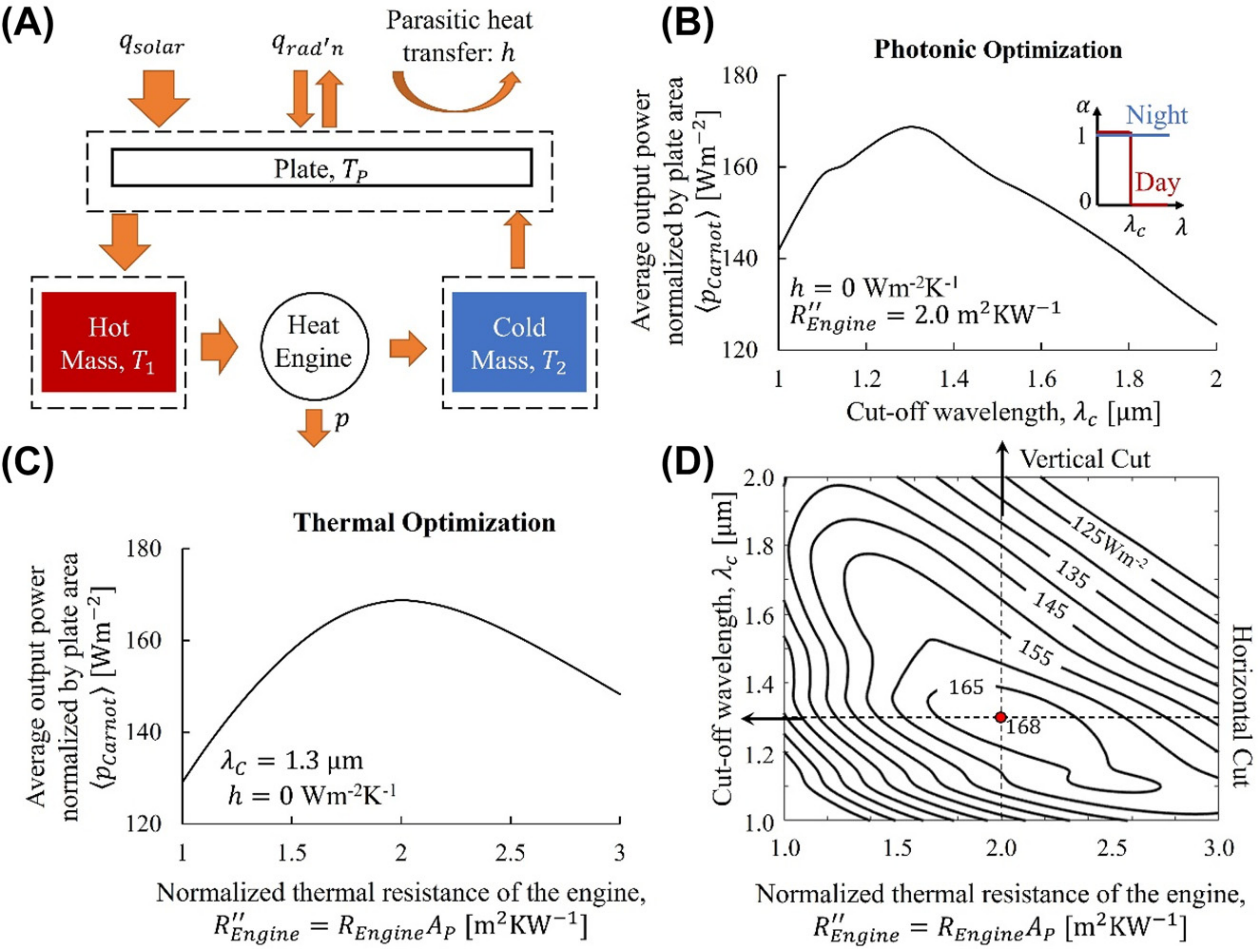
Coupled photonic and thermal optimization of the temperature-doubler circuit with the self-adaptive absorber/emitter (Figure 1B-III) under a realistic solar-thermal condition. (A) Energy balance of the plate and thermal masses. (B) Optimizing cut-off wavelength, *λ*_*C*_, to maximize the average output power normalized by the surface area of the plate, $\left\langle p_{\mathrm{Carnot}}\right\rangle$ , assuming fixed engine resistance normalized with plate area, $R_{\mathrm{Engine}}^{\prime \prime }$ . (C) Optimizing $R_{\mathrm{Engine}}^{\prime \prime }$ to maximize $\left\langle p_{\mathrm{Carnot}}\right\rangle$ , assuming fixed *λ*_*C*_. (D) Contour plot of $\left\langle p_{\mathrm{Carnot}}\right\rangle$ as a function of $R_{\mathrm{Engine}}^{\prime \prime }$ and *λ*_*C*_. This example assumes a Carnot engine and perfect thermal insulation of the temperature-doubler. Note the kinks in (B–D) originate from the AM1.5 solar spectrum (gray line in Figure 5B).

To describe the physics above, we consider the energy balance of the plate and the two thermal masses, as shown in Figure 2A, respectively,

(1) $$\begin{array}{ll} C_{P}\frac{dT_{P}}{dt} & =A_{P}\left[q_{\mathrm{solar}}-q_{rad^{\prime }n}-h\left(T_{P}-T_{\infty }\right)\right]\\ & \;-\frac{T_{P}-T_{1}}{R_{\mathrm{Diode,1}}}-\frac{T_{P}-T_{2}}{R_{\mathrm{Diode,2}}}, \end{array}$$

(2) $$C_{1}\frac{dT_{1}}{dt}=\left(\frac{T_{P}-T_{1}}{R_{\mathrm{Diode,1}}}-\frac{T_{1}-T_{2}}{R_{\mathrm{Engine}}}\right),$$

(3) $$C_{2}\frac{dT_{2}}{dt}=\left[\frac{T_{P}-T_{2}}{R_{\mathrm{Diode,2}}}+\frac{T_{1}-T_{2}}{R_{\mathrm{Engine}}}-P\right],$$

where *q*_*solar*_ is the solar flux absorbed by the plate (with SI units W m^−2^), which, in this work, is simplified to

(4) $$q_{\mathrm{solar}}=\left\{\begin{array}{cc} \int_{0}^{\infty }\alpha \left(\lambda \right)G_{\mathrm{AM1.5}}\left(\lambda \right)d\lambda \sin\left(\omega t+\phi_{\text{solar}}\right),\; & during\;the\;day\\ 0,\; & at\;night \end{array}\right.,$$

where $\alpha \left(\lambda \right)$ is the spectral absorptivity of the plate, *G*_*AM1,5*_ is the AM1.5 solar intensity (gray in Figure 5B) [28], the spectral integration of which gives $q_{0}=\int_{0}^{\infty }G_{\mathrm{AM1.5}}\left(\lambda \right)d\lambda =1000$ W m^−2^. In the simplified spectra, e.g. Figure 1B-II and III, the integration in Eq. (4) is truncated by a cut-off wavelength, *λ*_*C*_. Here the phase *ϕ*_*solar*_ is chosen so that *ωt* + *ϕ*_*solar*_ = 0 at sunrise.

(5) $$\begin{array}{ll} q_{rad^{\prime }n} & =\int d\Omega \cos\theta \int_{0}^{\infty }\varepsilon \left(\lambda ,\Omega \right)\left[I_{BB}\left(T_{P},\lambda \right)-\varepsilon_{\mathrm{atm\cdot}}\left(\lambda ,\Omega \right)\right.\\ & \;\times \left.I_{BB}\left(T_{\infty },\lambda \right)\right]d\lambda , \end{array}$$

is the net radiative heat transfer between the plate and environment, where $\int d\Omega =\int_{0}^{\pi /2}\sin\theta d\theta \int_{0}^{2\pi }\mathrm{d\varphi}$ is an integral over the hemisphere, $\varepsilon \left(\lambda ,\Omega \right)$ and $\varepsilon_{\mathrm{atm\cdot}}\left(\lambda ,\Omega \right)$ are the spectral directional emissivity of the plate and atmosphere (gray in Figure 5C) [28]. *I*_*BB*_ is the blackbody intensity.

Here we treat the thermal masses, *C*_*P*_, *C*_1_, and *C*_2_, as ‘‘lumped’’, i.e., uniform temperatures, *T*_*P*_, *T*_1_, and *T*_2_, within the plate and the masses at any instant in time. *h* is the parasitic heat transfer coefficient between the plate and the environment with temperature, *T*_∞_, which is simplified as

(6) $$T_{\infty }=T_{\mathrm{ref\cdot}}+T_{\mathrm{ampl\cdot}}\sin\left(\omega t+\phi_{\mathrm{amb.}}\right),$$

where *T*_*ref⋅*_ = 300 K and *T*_*ampl⋅*_ = 10 K. Consistent with typical conditions we assume that the peak air temperature occurs 2 h after solar noon, so that *ϕ*_*solar*_ − *ϕ*_*amb⋅*_ = *π*/6.

*R*_*Diode,1*_ and *R*_*Diode,2*_ are the instantaneous thermal resistances of the two thermal diodes. We model each thermal diode as perfectly switchable between a constant forward resistance, *R*_*F*_, and a constant reverse resistance, *R*_*B*_ [14]. For example, for the diode on the hot side, we have

(7) $$R_{\mathrm{Diode,1}}=\left\{\begin{array}{cc} R_{F},\; & T_{P}\geq T_{1}\\ R_{B},\; & T_{P}<T_{1} \end{array}\right..$$

Here we have three unknowns (*T*_1_, *T*_2_, and *T*_*P*_) and three constraints (Eqs. (1)–(3)), and thus this is a well-posted problem. Now mathematically the goal is to maximize the output power,

(8) $$P=p\cdot A_{p}=\frac{T_{1}-T_{2}}{R_{\mathrm{Engine}}}\times \eta ,$$

by optimizing the parameters (*C*_*P*_, *C*_1_, *C*_2_, *R*_*Diode,1*_, *R*_*Diode,2*_, *λ*_*C*_, *R*_*Engine*_, *A*_*P*_). Here *R*_*Engine*_ (SI units K W^−1^) is the thermal resistance of the heat engine, *p* is the output power, *P*, normalized by the plate area, *A*_*P*_, and *η* is the efficiency of the heat engine. While we have already considered (*C*_*P*_, *C*_1_, *C*_2_, *R*_*Diode,1*_, and *R*_*Diode,2*_) in our previous work [14], in this work we focus on the optimization of *λ*_*C*_ and $R_{\mathrm{Engine}}^{\prime \prime }=R_{\mathrm{Engine}}\cdot A_{p}$ , which are related to the photonic and the thermal design, respectively. As discussed in detail in Appendix B, instead of $R_{\mathrm{Engine}}^{\prime \prime }$ , we could choose ${\hat{R}}_{\mathrm{Engine}}=R_{\mathrm{Engine}}\cdot A_{p}\cdot h$ , a dimensionless Biot number defined in classic heat transfer analysis, which, however, is not convenient for the analysis of the ideal scenario of *h* → 0 (see, for example, Figure 2B–D).

To explore the theoretical limits of the temperature-doubler circuit, we assign Carnot efficiency,

(9) $$\eta =\eta_{\mathrm{Carnot}}=1-\frac{T_{2}}{T_{1}},$$

to the heat engine, and neglect the parasitic thermal exchange between the plate and the environment, i.e. *h* → 0. According to our previous work [14], we further assume infinite hot and cold thermal masses, *C*_1_ = *C*_2_ → ∞, ideal thermal diodes with *R*_*B*_ → ∞ and *R*_*F*_ → 0, and zero thermal mass of the plate *C*_*P*_ → 0 (see Appendix D for realistic plates with *C*_*P*_ > 0).

With these assumptions and parameters, we now optimize *λ*_*C*_ and $R_{\mathrm{Engine}}^{\prime \prime }$ . To illustrate the optimization process, we use the self-adaptive absorber/emitter as a concrete example. First, we fix $R_{\mathrm{Engine}}^{\prime \prime }=R_{\mathrm{Engine}}\cdot A_{p}=2.0$ m^2^ K W^−1^ and optimize *λ*_*C*_. At daytime, while solar absorption prefers a larger *λ*_*c*_, the infrared thermal insulation favors a smaller *λ*_*c*_. As shown in Figure 2B, this competition leads to an optimal *λ*_*c*_ to maximize the power output (normalized by *A*_*p*_) averaged in a cycle, $\left\langle p_{\mathrm{Carnot}}\right\rangle$ . Next, we fix *λ*_*C*_ = 1.3 μm and optimize $R_{\mathrm{Engine}}^{\prime \prime }$ . While a zero $R_{\mathrm{Engine}}^{\prime \prime }$ leads to zero temperature difference across the heat engine and thus zero efficiency, an infinite $R_{\mathrm{Engine}}^{\prime \prime }$ leads to zero heat flow through the heat engine and thus zero power output. Therefore, there must exist an optimal $R_{\mathrm{Engine}}^{\prime \prime }$ to maximize $\left\langle p_{\mathrm{Carnot}}\right\rangle$ (Figure 2C). Combining these two optimizations leads to the contour plot in Figure 2D and the maximum average output, $\left\langle p_{\mathrm{Carnot,max}}\right\rangle =168$ W m^−2^.

**Figure 3:**
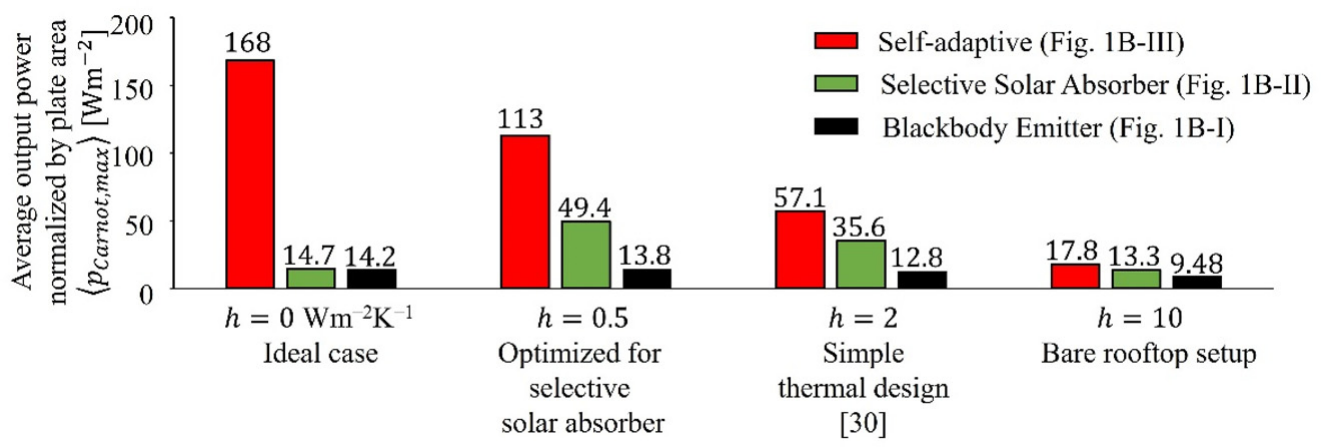
Maximum output power, $\left\langle p_{\mathrm{Carnot,max}}\right\rangle$ , of the temperature-doubler circuit (see Table 1 for the corresponding optimized photonic and thermal parameters, *λ*_*C*_ and $R_{\mathrm{Engine}}^{\prime \prime }$ ) under various scenarios. In their optimized scenarios, the self-adaptive absorber/emitter (red) generates 12 and 3.4 times more power than the blackbody emitter (black) and the selective solar absorber (green), respectively. The maximum $\left\langle p_{\mathrm{Carnot,max}}\right\rangle$ of the selective solar absorber appears at *h* = 0.5 W m^−2^ K^−1^ instead of *h* = 0 W m^−2^ K^−1^, because this spectrum has trouble to be cooled down at night in the latter scenario, as shown in Figure 1D. We note that the high plate temperature in Figure 1D–E corresponds to *h* = 0 W m^−2^ K^−1^. In practical scenarios with *h* = 10 W m^−2^ K^−1^ (a bare rooftop setup) or *h* = 2 W m^−2^ K^−1^ (a simple thermal design), the plate temperature is 400 K.

With the same optimization process, we now compute $\left\langle p_{\mathrm{Carnot,max}}\right\rangle$ of the temperature-doubler circuit with the three simplified spectra (Figure 1B) under representative scenarios characterized by the parasitic heat transfer coefficient, *h* (Figure 3), including the ideal case (*h* = 0 W m^−2^ K^−1^), a simple thermal design (*h* = 2 W m^−2^ K^−1^) [29], and a bare rooftop setup (*h* = 10 W m^−2^ K^−1^). We also consider a case of *h* = 0.5 W m^−2^ K^−1^, corresponding to the maximum $\left\langle p_{\mathrm{Carnot,max}}\right\rangle$ of the selective solar absorber, which is distinct from the other two spectra. Regardless of the values of *h*, the self-adaptive absorber/emitter (red) always outperforms the other two static photonic designs. It is worth noting that while for the self-adaptive (red) and the blackbody emitter (black), $\left\langle p_{\mathrm{Carnot,max}}\right\rangle$ monotonically decreases with the increase of *h*, there is an optimal *h* for the selective solar absorber (green). This is because, without the parasitic heat dissipation mechanism, the selective solar absorber struggles to cool down the cold mass at night, which is evident from Figure 1D. These comparisons highlight that, in their optimized scenarios, the self-adaptive absorber/emitter generates 12 times (3.4 times) more power than the blackbody emitter (selective solar absorber). We note that all these scenarios are optimized separately, with different optimal ( $R_{\mathrm{Engine}}^{\prime \prime },\lambda_{c}$ ) pairs (Table 1).

**Table 1:** Optimal photonic and thermal parameters, *λ*_*C*_ and $R_{\mathrm{Engine}}^{\prime \prime }$ , corresponding to the maximum output power, $\left\langle p_{\mathrm{Carnot,max}}\right\rangle$ , of the temperature-doubler circuit under various scenarios in Figure 3.

$\left(R_{\mathrm{Engine}}^{\prime \prime },\lambda_{c}\right)$		Self-adaptive	Selective Solar	Blackbody
$\left[{\text{m}}^{2\;}\text{K}{\text{W}}^{-1},\;\mu m\right]$		(Figure 1B-III)	Absorber	Emitter
			(Figure 1B-II)	(Figure 1B-I)
Parasitic	0	(2.0, 1.3)	(0.8, 5.9)	(0.6, N/A)
heat transfer	0.5	(1.5, 1.8)	(5.1, 1.1)	(0.5, N/A)
coefficient,	2	(1.0, 2.4)	(1.9, 2.3)	(0.5, N/A)
*h* [W m^−2^ K^−1^]	10	(0.4, 2.5)	(0.5, 2.5)	(0.3, N/A)

### 2.3 Comparison with traditional thermal circuits

**Figure 4:**
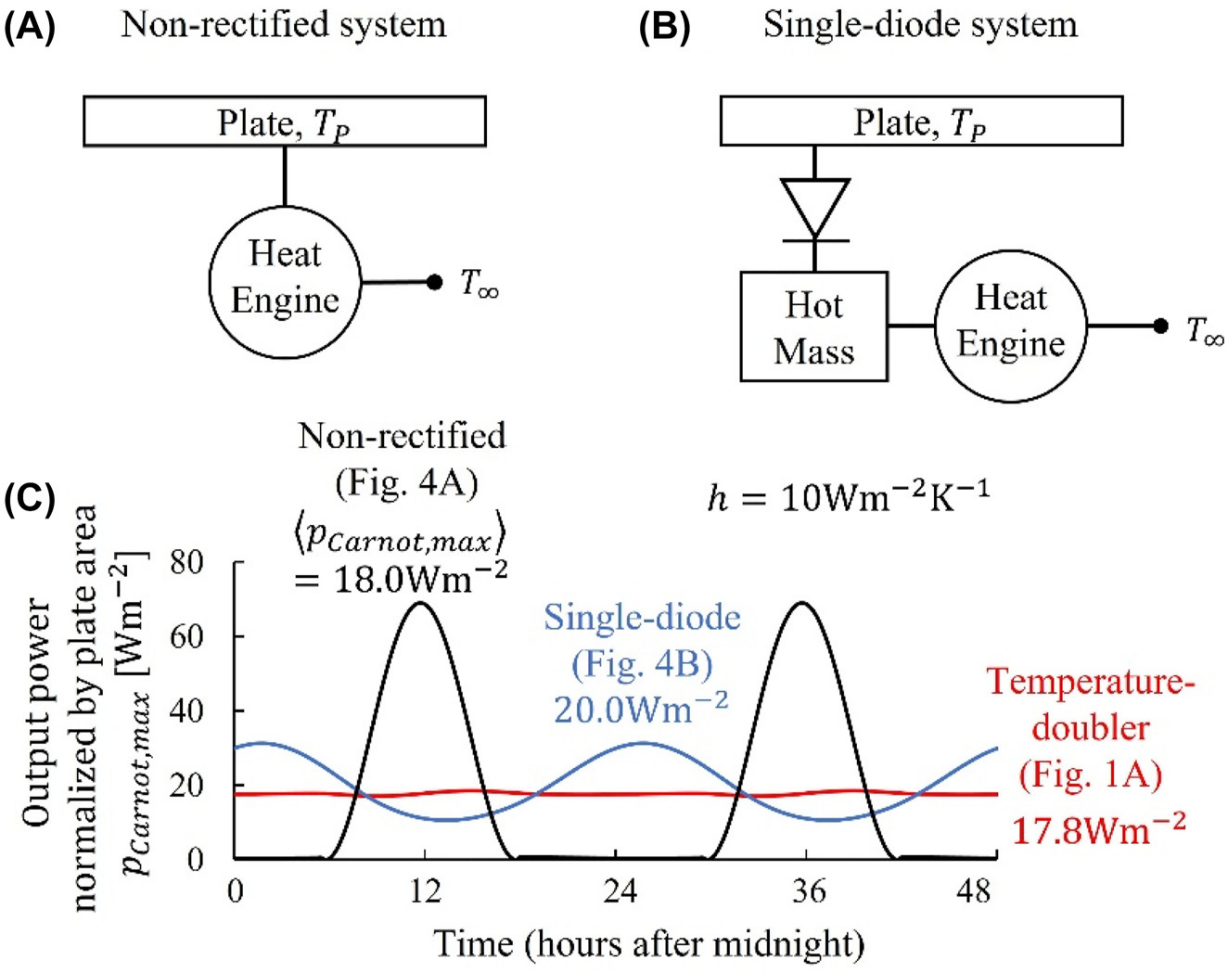
Comparison with (A) a non-rectified waste heat scavenging system and (B) a solar thermal power generation system. (C) Output power as a function of time, for the configurations in (A) (black) and (B) (blue) here and Figure 1A (red) under the realistic solar-thermal boundary condition. Here we assume a self-adaptive absorber (Figure 1B–III), a Carnot engine, and a typical rooftop parasitic heat transfer coefficient (*h* = 10 W m^−2^ K^−1^). As elsewhere in this work, the ( $R_{\mathrm{Engine}}^{\prime \prime },\lambda_{c}$ ) pairs are optimized for the three configurations separately. These comparisons highlight that the main advantage of the temperature-doubler circuit is to wash out the fluctuation of the power output from the periodic thermodynamic input.

We now compare the performance of the temperature-doubler circuit to that of a non-rectified system (Figure 4A), which has neither diode nor thermal mass. This configuration closely resembles a basic waste heat scavenging application. Here we assume the heat engines is bipolar, i.e. it functions either when *T*_*P*_ > *T*_∞_ or when *T*_*P*_ < *T*_∞_, which means it can operate over the full period.

We have demonstrated experimentally that, for the idealized sinusoidal temperature resource, the temperature-doubler circuit (Figure 1A) generates four times more power than the non-rectified system (Figure 4A), with a theoretical limit of an 8-fold enhancement for perfect thermal diodes and large thermal masses [14]. A natural question here is whether or not a similar conclusion holds for a more realistic solar-thermal boundary condition considered in this work.

To answer this question, we modify the energy balance of the plate from Eq. (1) for the temperature-doubler circuit (Figure 1A) to the following equation for the non-rectified system (Figure 4A),

(10) $$C_{P}\frac{dT_{P}}{dt}=A_{P}\left[q_{\mathrm{solar}}-q_{rad^{\prime }n}-h\left(T_{P}-T_{\infty }\right)\right]-P,$$

where the output power

(11) $$P=\frac{T_{P}-T_{\infty }}{R_{\mathrm{Engine}}}\times \eta .$$

with this model (Eqs. (10) and (11)) validated by published works, e.g. Refs. [17], [30], [31], we now compare the output of the two circuits, both equipped with the self-adaptive absorber/emitter (Figure 1B-III). With the same assumptions and input parameters as those in last section, Figure 4C shows the comparison between the temperature-doubler circuit (Eqs. (1)–(3) and (8)) and the non-rectified system (Eqs. (10) and (11)), both with a Carnot engine (Eq. (9)). In contrast to the fluctuating output of the non-rectified system (black in Figure 4C), the most important takeaway of the temperature-doubler circuit (red) is the “DC” power output in a 24 h day-night cycle, which is expected from the temperature profile in Figure 1C–E and agrees with our previous work [14].

A distinct feature as compared to the results with the idealized sinusoidal temperature boundary condition [14], however, is that the maximal average power output of the two circuits, optimized to different ( $R_{\mathrm{Engine}}^{\prime \prime },\lambda_{c}$ ) pairs, respectively, are comparable. This is because the ideal temperature resource is a “voltage source” without output impedance, and thus it can source and sink arbitrarily large heat flows without any penalty; but the realistic solar-thermal resource is a “current source” with a maximal incoming heat current of *q*_0_ ≈ 1000 W m^−2^ under the peak solar irradiance, or a maximal outgoing heat current of *q*_*rad*′*n*_ ≈ 150 W m^−2^ under a clear night sky.

Table 2 summarizes more comparisons among various scenarios. Three features are worth noting. First, for the self-adaptive absorber, the temperature-doubler circuit gradually loses the competition, as the parasitic heat transfer coefficient, *h*, increases from 0 to 2 W m^−2^ K^−1^. Second, as *h* increases further to 20 W m^−2^ K^−1^, however, the competition prefers the temperature-doubler again. This is because in the limiting case of *h* → ∞, this solar-thermal boundary condition recovers the ideal sinusoidal temperature boundary condition, although in this limit the driving force is the fluctuation of the ambient temperature (Eq. (6)). Recall our conclusion with the temperature boundary condition that the temperature-doubler circuit generates more power than the traditional circuit [14]. The last feature is that for a selective solar absorber with a good insulation design of the plate (*h* = 0.5 W m^−2^ K^−1^), the temperature-doubler loses significantly, because in this case it does not have an efficient heat dissipation mechanism at night, which is as evident from Figure 1D; on the other hand, the non-rectified system (Figure 4A) overcomes this severe limitation by anchoring the cold side of the engine to *T*_∞_. Therefore, under the more realistic solar-thermal boundary condition, the main advantage of the temperature-doubler circuit is not to enhance the output power, but to even out the fluctuation (Figure 4C).

**Table 2:** A summary of the comparison between the temperature-doubler circuit and the non-rectified system with the simplified spectra and various parasitic heat transfer coefficients, all with a Carnot engine. All these scenarios are optimized separately following the procedure in Section 2.2.

Spectrum	Parasitic	Average output normalized by plate area,	
	heat transfer	$\left\langle p_{\mathrm{Carnot,max}}\right\rangle$ [W m^−2^]	
	coefficient, *h* [W m^−2^ K^−1^]	Temperature-doubler (Figure 1A)	Non-rectified system (Figure 4A)
Self-adaptive	0	168	161
Absorber/Emitter	2	57.1	62.3
(Figure 1B-III)	20	11.5	9.54
Selective Solar			
Absorber	0.5	49.4	116
(Figure 1B-II)			

We close this section by briefly comparing the temperature-doubler circuit to a solar thermal power generation system [32], in which the working fluid and its storage tank, e.g. the molten salt, serves the function of the thermal mass. We also note that the working fluid receives heat from the solar collector only in the daytime but not at nighttime, very similar to the behavior of a thermal diode. Figure 4B describes the essential functionalities of this solar thermal power generation system, which consists of one diode and one thermal mass on the hot side, with the cold side fixed at the ambient temperature, *T*_2_ = *T*_∞_. With the same input parameters, Figure 4C shows much smoother power output of this solar thermal power generation system (blue) as compared to that of the non-rectified system (black), due to the diode and the mass on the hot side (Figure 4B); however, due to the lack of the diode and the mass on the cold side to smooth the fluctuation of *T*_∞_ (Eq. (6)), the output of the solar thermal power generation system (blue) is not as stable as that of the temperature-doubler circuit (red).

### 2.4 A photonic design of the self-adaptive absorber/emitter

At last, we provide a photonic design of the self-adaptive absorber/emitter to utilize the full potential of the sun and outer space for energy harvesting. With this photonic design, we generate synthetic “experimental” data under realistic conditions to demonstrate the merits of this self-adaptive feature.

**Figure 5:**
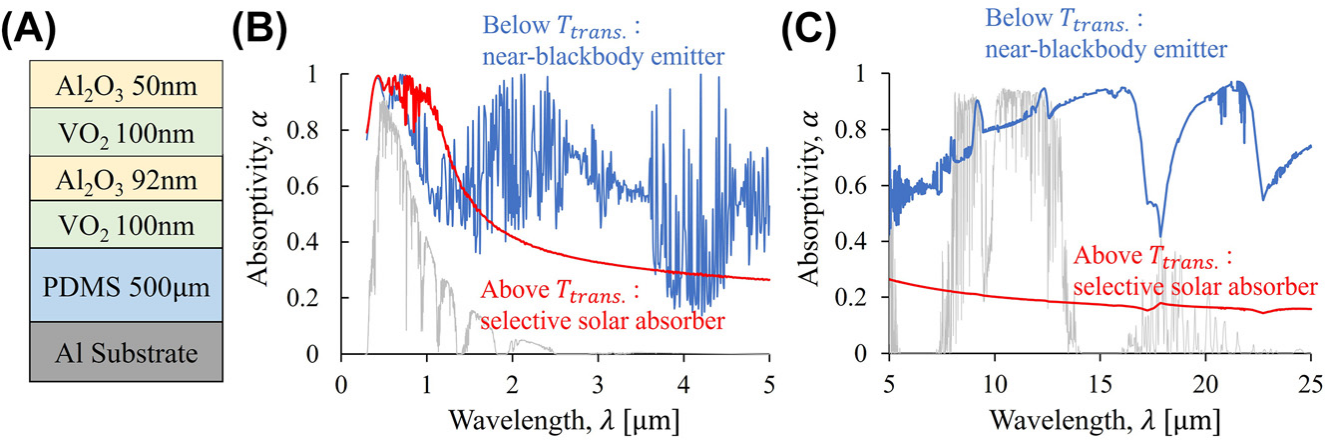
A photonic design of the self-adaptive absorber/emitter. (A) Schematic cross-sectional view of the multilayer stack. (B–C) Calculated spectral absorptivity spectrum of the self-adaptive photonic design along the normal direction, with the AM1.5 solar spectrum (gray in B) and a typical atmospheric transmittance (gray in C) [28] as references. This design takes advantage of the phase transition of VO_2_, which switches between a selective solar absorber above the transition temperature, *T*_*trans⋅*_, and a near-black emitter below *T*_*trans⋅*_.

**Figure 6:**
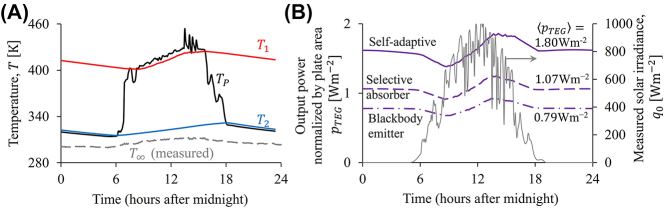
Synthetic “experiments” to demonstrate the advantages of the photonic design (Figure 5) with the temperature-doubler circuit and a thermoelectric generator (TEG). (A) Simulated temperatures of the plate, *T*_*P*_ (black), and the two masses, *T*_1_ (red), and *T*_2_ (blue), with measured ambient temperature, *T*_∞_ (gray), and solar irradiance, *q*_0_ (gray in (B), as input parameters to Eqs. (1)–(3), (7), (8), and (12). (B) Output power of the temperature-doubler circuit with the photonic design in Figure 5A (solid purple line) and two static spectra (see text).

Here the key to realize the self-adaptivity of the spectrum is to utilize the phase transition of vanadium dioxide (VO_2_), which behaves as a metal (insulator) above (below) the transition temperature, *T*_*trans⋅*_ [22], [33]–[38]. Varying the doping level of tungsten (W) and strontium (Sr) atoms in intrinsic VO_2_, one can vary *T*_*trans⋅*_ in a wide range from 341 K to below 300 K [35], [36], [38]. Here we choose *T*_*trans⋅*_ = 330 K to facilitate the switch between a selective solar absorber and an infrared emitter at sunset and sunrise (Figure 6A). Our photonic design consists of six layers, which are made of VO_2_ and other two common materials, aluminum oxide (Al_2_O_3_) and polydimethylsiloxane (PDMS), and an aluminum layer (Al) as the back mirror (Figure 5A). Above *T*_*trans⋅*_, this design is a selective solar absorber (red lines in Figure 5B and C) with high absorptivity in the wavelength range of 0.3–1.1 μm, while below *T*_*trans⋅*_, it turns to a near-blackbody emitter in the full spectrum of interest (blue lines in Figure 5B and C). The frequency-dependent refractive index of VO_2_ and other materials are from Refs. [22], [34], [37], [39]–[41].

To be more realistic, we consider a thermoelectric generator (TEG) with efficiency

(12) $$\eta =\beta \cdot \eta_{\mathrm{Carnot}}=\beta \cdot \left(1-\frac{T_{2}}{T_{1}}\right),$$

where $\beta =\left(\sqrt{1+ZT}-1\right)/\left(\sqrt{1+ZT}+T_{2}/T_{1}\right)$ [42]. In this work we choose the dimensionless figure-of-merit *ZT* = 1 to represent commercial TEGs.

We now discuss the experimental design sequence. First, we fix the parasitic heat transfer coefficient, *h* = 0.2 W m^−2^ K^−1^, corresponding to a vacuum-level (∼10^−6^ Torr) thermal insulation [43]. We note that in this scenario the parasitic heat leakage is dominated by the thermal radiation between the backside of the plate and the upper surface of the thermal masses. To reach this level of thermal insulation, we require an effective emissivity, *ɛ*_*eff⋅*_ = 0.04, of the mass surfaces and the backside of the plate, according to a rough estimate of $h_{rad^{\prime }n}\approx 4\varepsilon_{\mathrm{eff\cdot}}\sigma T_{\mathrm{avg.}}^{3}/2$ [43] and the calculated *T*_*avg⋅*_ = 365 K that is averaged between the temperature of the plate and masses (Figure 6A). Such low effective emissivity can be achieved by highly polished metal surfaces. Second, with *h* and the photonic design fixed, we conduct the thermal optimization (Figure 2C) to find the optimal $R_{\mathrm{Engine}}^{\prime \prime }=1.9$ m^2^ K W^−1^, which gives *R*_*Engine*_ = 1.9 KW^−1^ if assuming *A*_*p*_ = 1 m^2^. At last, according to our previous optimization [14], which requires $\hat{C}=R_{\mathrm{Engine}}C/\tau \geq 5$ , ${\hat{R}}_{B}=R_{B}/R_{\mathrm{Engine}}\geq 1$ , and ${\hat{R}}_{F}=R_{F}/R_{\mathrm{Engine}}\leq 0.01$ to maximize the output power, we choose *C*_1_ = *C*_2_ = 2.3 × 10^5^ JK^−1^, *R*_*B*_ = 1.9 K W^−1^, and *R*_*F*_ = 0.019 K W^−1^. As a practical example, this choice of heat capacity corresponds to 110 kg (0.11 m^3^) water.

With these design parameters, we now perform virtual “experiments” under typical conditions in summer in Nanjing, China. With measured *T*_∞_ (gray in Figure 6A) and *q*_0_ (gray in Figure 6B) on Jun. 28, 2023, one computes the corresponding *T*_*P*_ (black in Figure 6A), *T*_1_ (red), *T*_2_ (blue), and *p*_*TEG*_ (purple in Figure 6B) using Eqs. (1)–(3), (5), (7), (8), and (12). In addition to the advantages we have already discussed, Figure 6 reveals another merit of the temperature-doubler circuit: although *T*_*P*_ fluctuates heavily due to the random fluctuation of the solar irradiance, *q*_0_, this fluctuation is washed out by the temperature-doubler circuit, and thus does not appear in *T*_1_, *T*_2_, and the final output power, *p*_*TEG*_. As compared to the blackbody emitter (dash line) and the static photonic design (dot-dashed line, spectrum fixed to the red one in Figure 5B for both day and night), each with their own optimized design parameters, the self-adaptive photonic design generates 2.3 and 1.7 times more power (Figure 6B).

## 3 Conclusions and discussion

To fully exploit the thermodynamic potential of both the sun and outer space, we combined the temperature-doubler circuit with a self-adaptive absorber/emitter to take advantage of the static selective solar absorber and the blackbody emitter while avoiding their demerits. We have developed a general framework for the coupled photonic and thermal optimization to maximize the power output of the temperature-doubler under a realistic solar-thermal boundary condition. Under perfect thermal insulation between the temperature-doubler and its environment, this optimization sets the upper bound of the average power output in a diurnal cycle, 168 W m^−2^, for a Carnot heat engine with ideal thermal diodes and masses. As compared to traditional thermal circuits, we showed that, under the realistic solar-thermal boundary condition, the major advantage of the temperature-doubler is to generate a stable and continuous power output from the periodic thermodynamic input. Building upon the phase transition of VO_2_, we proposed a simple multilayer stack to realize such a self-adaptive photonic design of the plate. With a more realistic thermoelectric generator, we performed “experiments” to demonstrate the advantage of the self-adaptive photonic design over its corresponding static design and a blackbody emitter.

We end by briefly commenting on the experimental implementation of such a temperate-doubler circuit. Common thermal storage materials such as water or metals [44] can be used for thermal masses. Among other heat engines, e.g. Stirling and Rankine, the thermoelectric generator, although less efficient, has advantages such as its reliability and ease of integration, and is thus a good choice of the heat engine for a proof-of-concept experimental demonstration. Thermal diodes/thermal switches have been recently demonstrated with diodicity/switch ratios of ∼10–100 [10], [45], [46]. In particular, paraffin actuators [47] or memory-alloy based thermal switches [10] are strong candidates for such applications. The challenge here is to make high-performance and reliable self-adaptive absorber/emitter. To maximize power output, one requires vacuum-level thermal insulation of the absorber/emitter to achieve high temperatures at daytime (Figure 1E). The vacuum level required here, ∼ 10^−6^ Torr, is similar to that used in commercial evacuated solar water collectors [48], and thus is not an issue. The reliability of the adaptive emitter at temperatures up to ∼ 800 K (Figure 1E), however, will be a key challenge. In addition, the parasitic heat loss at such high temperatures will also require extra care. We envision future efforts along this direction to ensure implementation of high-performance and reliable temperature-doubler circuit with self-adaptive absorber/emitter.

## Figures and Tables

**Figure A.1:**
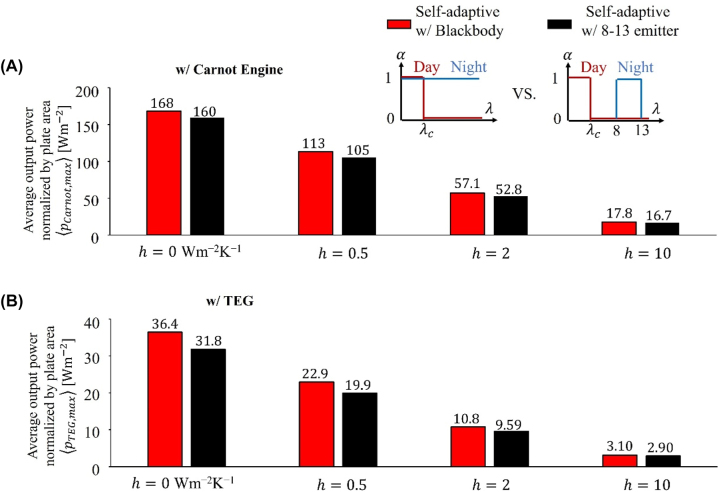
Comparison between the nighttime spectrum of the self-adaptive absorber/emitter: blackbody versus selective emitter, with (A) a Carnot engine and (B) a thermoelectric generator (TEG). Blackbody emitter wins the competition regardless of the engine and *h*.

**Figure A.2:**
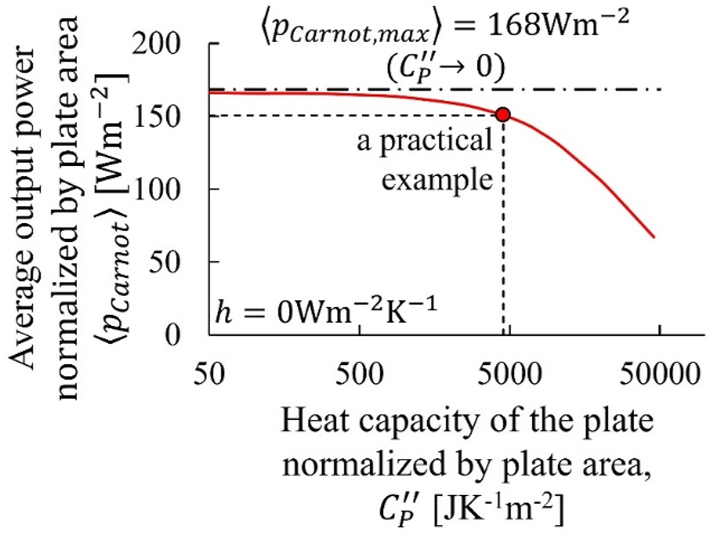
Realistic plates with nonzero *C*_*P*_. To be consistent with the calculations in Section 2.2, here we assume the ideal self-adaptive photonic design (Figure 1B–III) and a Carnot engine, with *h* = 0 W m^−2^ K^−1^, *C*_1_ = *C*_2_ → ∞, *R*_*B*_ → ∞, and *R*_*F*_ → 0.

## Appendix A: Nomenclature

**Latin letters**
*A*_*P*_: surface area of the plate
*C*_*P*_, *C*_1_, *C*_2_: heat capacity of the plate, hot mass, and cold mass
*C*″: heat capacity normalized by the plate area: *C*″ = *C*/*A*_*P*_
$\hat{C}$: dimensionless heat capacity: $\hat{C}=R_{\mathrm{Engine}}C/\tau$
*h*: parasitic heat transfer coefficient
*h*_*rad*′*n*_: parasitic parasitic heat transfer coefficient due to radiation between the plate and the thermal masses
*G*_*AM1.5*_: AM1.5 solar intensity
*I*_*BB*_: blackbody radiation intensity
*P*: output power of the heat engine, with SI units W
*p*: *P* normalized by plate area, with SI units W m^−2^
*p*_*Carnot*_: *p* of a Carnot engine with ideal thermal diodes and masses
*p*_*TEG*_: *p* of a thermoelectric generator
*p*_max_: maximum *p* through coupled photonic and thermal optimization
*q*_*solar*_: solar flux absorber by the plate, with SI units W m^−2^
*q*_0_: solar constant
*q*_*rad*′*n*_: radiative heat flux between plate and environment
*R*_*Engine*_: thermal resistance of the heat engine, with SI units KW^−1^
*R*_*Diode,1*_, *R*_*Diode,2*_: thermal resistance of thermal diodes
*R*_*B*_, *R*_*F*_: backward and forward thermal resistance of the thermal diode
*R*″: resistance normalized by plate area: *RA*_*P*_, with SI units m^2^ K W^−1^
$\hat{R}$: dimensionless thermal resistance: $\hat{R}=R/R_{\mathrm{Engine}}$
*T*_*P*_, *T*_1_, *T*_2_, *T*_∞_: temperature of the plate, hot mass, cold mass, and environment
*T*_*ref⋅*_, *T*_*ampl.*_: mean value and amplitude of *T*_∞_ (Eq. (6))
*T*_*avg⋅*_: average temperature between the plate and the mass
Δ*T*_*Engine*_: temperature difference between hot and cold thermal masses
*t*: time
*ZT*: thermoelectric figure-of-merit

**Greek letters**
*α*: absorptivity
*β*: proportional factor in Eq. (12)
*ɛ*: emissivity
*ɛ*_*atm⋅*_: emissivity of the atmosphere
*ɛ*_*eff⋅*_: effective emissivity
*η*: efficiency of heat engine
*θ*, *φ*: incident angle
*λ*: wavelength
*λ*_*C*_: cut-off wavelength of the spectrum
*σ*: Stefan–Boltzmann constant (5.67 × 10^−8^ W m^−2^ K^−4^)
*τ*: period of a diurnal cycle
*ϕ*_*solar*_, *ϕ*_*amb⋅*_: phase of the periodic solar irradiance and ambient temperature
*ω*: angular frequency
Ω: solid angle

**Operators**
⟨⟩: time average: $X=\frac{1}{\tau }\int_{t_{0}}^{t_{0}+\tau }X\left(t\right)dt$

## Appendix B: Re-arranging Eqs. (1)–(3)

Equations (1)–(3) in the main text can be re-written as

$$\begin{array}{ll} C_{P}^{\prime \prime }\frac{dT_{P}}{dt} & =q_{\mathrm{solar}}-q_{rad^{\prime }n}-h\left(T_{P}-T_{\infty }\right)-\frac{T_{P}-T_{1}}{R_{\mathrm{Diode,1}}^{\prime \prime }}\\ & \;-\frac{T_{P}-T_{2}}{R_{\mathrm{Diode,2}}^{\prime \prime }}, \end{array}$$

$$C_{1}^{\prime \prime }\frac{dT_{1}}{dt}=\left(\frac{T_{P}-T_{1}}{R_{\mathrm{Diode,1}}^{\prime \prime }}-\frac{T_{1}-T_{2}}{R_{\mathrm{Engine}}^{\prime \prime }}\right),$$

$$C_{2}^{\prime \prime }\frac{dT_{2}}{dt}=\left[\frac{T_{P}-T_{2}}{R_{\mathrm{Diode,2}}^{\prime \prime }}+\frac{T_{1}-T_{2}}{R_{\mathrm{Engine}}^{\prime \prime }}-P\right],$$

where $C^{\prime \prime }=\frac{C}{A_{P}}$ (with SI units J K^−1 ^m^−2^) and *R*″ = *R*⋅*A*_*P*_ (with SI units m^2^ K W^−1^) are the heat capacity and the thermal resistance of the engine, both normalized by the surface area of the plate.

Instead of $R_{\mathrm{Engine}}^{\prime \prime }$ , we could choose ${\hat{R}}_{\mathrm{Engine}}=R_{\mathrm{Engine}}\cdot A_{p}\cdot h$ , a dimensionless Biot number defined in classic heat transfer analysis, which, however, is not convenient for the analysis of the ideal scenario of *h* → 0 (see, for example, Figure 2B–D).

## Appendix C: Blackbody or selective emitter at night?

Unlike the application of radiative cooling at night, which prefers a selective emitter that emits only between 8 and 13 μm [43], here Figure A.1 shows that the self-adaptive absorber/emitter likes a blackbody emitter at night regardless of the type of the engine and the value of *h*. This is because the plate temperature, *T*_*P*_, is higher than the ambient temperature, *T*_∞_, at night, which means the blackbody emitter dissipates more infrared radiation than the selective emitter at night.

## Appendix D: Effect of the plate capacity

We consider realistic plates with nonzero heat capacity, *C*_*P*_. Qualitatively, as *C*_*P*_ increases, it becomes harder for the plate to reach its maximal and minimal temperatures of the ideal *C*_*P*_ → 0 case for a fixed diurnal cycle. As a result, the power output of the temperature-doubler circuit decreases with the increase of *C*_*P*_. Quantitatively, we follow the analysis in Section 2.2 to show this effect (Figure A.2).

As a concreate example, an aluminum plate with dimensions of 1 m^2^ × 2 mm has $C_{P}^{\prime \prime }=C_{P}/A_{P}=4752$ J K^−1^ m^−2^. Following the same assumptions as in Section 2.2, i.e. ideal self-adaptive photonic design (Figure 1B-III), a Carnot engine, *h* = 0 W m^−2^ K^−1^, *C*_1_ = *C*_2_ → ∞, *R*_*B*_ → ∞, and *R*_*F*_ → 0, this realistic plate leads to a 10 % decrease of the output power as compared to the ideal case of $C_{P}^{\prime \prime }=0$ (dot-dashed line in Figure A.2).
